# Curcumin and Vitamin C Attenuate Gentamicin-Induced Nephrotoxicity by Modulating Distinctive Reactive Species

**DOI:** 10.3390/metabo13010049

**Published:** 2022-12-28

**Authors:** Anamaria Magdalena Tomşa, Andreea Liana Răchişan, Stanca Lucia Pandrea, Andreea Benea, Ana Uifălean, Corina Toma, Roxana Popa, Alina Elena Pârvu, Lia Monica Junie

**Affiliations:** 1 2nd Pediatrics Clinic, ‘Iuliu Hatieganu’ University of Medicine and Pharmacy, 400177 Cluj-Napoca, Romania; 2 Department of Microbiology, ‘Iuliu Hatieganu’ University of Medicine and Pharmacy, 400012 Cluj-Napoca, Romania; 3 ‘Prof. Dr. Octavian Fodor’ Regional Institute of Gastroenterology and Hepatology, 400162 Cluj-Napoca, Romania; 4 Department of Pathophysiology, ‘Iuliu Hatieganu’ University of Medicine and Pharmacy, 400012 Cluj-Napoca, Romania; 5 Department of Veterinary Pathology, University of Agricultural Sciences and Veterinary Medicine, 400372 Cluj-Napoca, Romania

**Keywords:** gentamicin nephrotoxicity, oxidative stress, antioxidant, lipid peroxidation, malondialdehyde, nitric oxide, curcumin, vitamin C, nephroprotection

## Abstract

Gentamicin remains widely used in all age groups despite its well-documented nephrotoxicity; however, no adjuvant therapies have been established to counteract this side effect. Our study aimed to experimentally determine whether curcumin and vitamin C have nephroprotective effects and whether certain reactive species could be used as markers of early gentamicin nephrotoxicity. Wistar adult male rats were evenly distributed into four groups: control, gentamicin, curcumin and gentamicin, vitamin C and gentamicin (gentamicin: 60 mg/kg/day, intraperitoneally, 7 days). We determined renal function (urea, creatinine), oxidative stress (malondialdehyde, nitric oxide, 3-nitrotyrosine, total oxidative stress), and antioxidant and anti-inflammatory status (thiols, total antioxidant capacity, interleukin-10). Nephrotoxicity was successfully induced, as shown by the elevated creatinine levels in the gentamicin group. In contrast, supplementation with curcumin and vitamin C prevented an increase in urea levels while decreasing total oxidative stress levels compared to the gentamicin group. Moreover, vitamin C and curcumin distinctively modulate the levels of nitric oxide and malondialdehyde. Histological analysis showed more discrete lesions in rats that received vitamin C compared to the curcumin group.

## 1. Introduction

Gentamicin, a potent aminoglycoside antibiotic, was approved for medical use more than 5 decades ago and remains widely used, especially in moderate and severe infections with Gram-negative bacteria, despite its well-known nephrotoxic effects [1]. Gentamicin is eliminated primarily unmetabolized by glomerular filtration, and up to 25% of patients receiving it develop secondary nephrotoxicity [2]. Although numerous studies have been conducted to determine the precise mechanisms leading to this unfavorable outcome, specific adjuvant therapies have not yet been established [3].

Oxidative stress (OS) is primarily represented by reactive species of oxygen and nitrogen and numerous free radicals that, together, overcome the total endogenous antioxidant capacity, causing damage at various cellular levels [4,5]. OS has been shown to play a key role in the pathogenesis of gentamicin nephrotoxicity [6]; therefore, certain reactive species might be used both as markers of early kidney injury and as targets for complementary therapies [7]. However, despite extensive research in this area, no specific markers have been implemented in clinical practice.

Exogenous antioxidants are substances found in various vegetables, fruits and whole grains, as food supplements or even as medicines, and they are used as agents that prevent or ameliorate diseases triggered by OS [8,9].

Turmeric is an Indian spice obtained from the rhizome of *Curcuma longa* and its main constituents are curcuminoids, natural polyphenols. The main compound is curcumin (75–80%), which was first isolated in 1815. Curcumin is a water-insoluble powder identified as diferuloylmethane or 1,6-heptadiene-3,5-dione-1,7-bis(4-hydroxy-3-methoxyphenyl)-(1E,6E) [10]. Curcumin has keto-enol tautomers, with the enol form predominating in alkaline solutions and the keto form predominating in neutral and acidic solutions [11]. Extensive research has been conducted to determine and evaluate its antioxidant and antiproliferative effects, but standard therapeutic strategies have not yet been implemented in humans [12].

Vitamin C (ascorbic acid) is a micronutrient with an essential role in immune defense that cannot be synthesized by the human body [13]. It acts by scavenging free radicals and reducing them to water while subsequently turning into its oxidized form (dehydroascorbic acid), which is relatively unreactive and stable [14,15,16]. Research on the benefits of vitamin C covers a wide variety of diseases, from the common cold [17] to sepsis [18] and cancer [19]. Both curcumin and vitamin C can be found in various dietary supplements, while vitamin C has also been implemented as a prescription drug with oral and parenteral administration.

The main objective of our research was to experimentally determine whether curcumin and vitamin C have nephroprotective effects by assessing the relationship between systemic oxidative stress and total antioxidant capacity in rats with a mild form of renal injury induced by gentamicin. Furthermore, we aimed to determine whether specific reactive species could be used as markers of early gentamicin nephrotoxicity.

## 2. Materials and Methods

### 2.1. Animals

A total of twenty-eight adult male Wistar rats (Rattus norvegicus) weighing 310 g to 538 g were used in this study. The animals were obtained from the Animal Department of “Iuliu Hatieganu” University of Medicine and Pharmacy Cluj-Napoca. The animals were placed in standard polypropylene cages in a room with 40–50% humidity, 12/12-h light/dark cycles, at controlled temperature, at the Pathophysiology Department of the same institution. They were given unlimited access to food (standard pellets made by Cantacuzino National Military Medical Institute for Research and Development, Bucharest, Romania) and water. No enrichment was used for this experiment. The bedding was changed daily.

### 2.2. Experimental Protocol

The animals were arbitrarily distributed into four groups (n = 7 rats/group): control, gentamicin, curcumin and vitamin C. We preferred not to include a group of curcumin only and a group of vitamin C only to minimize the number of animals used. The number of animals in each group was reduced to the minimum necessary to obtain satisfactory statistical power, even in the event of a premature death. The total time of the experiment was 10 days.

Rats in the control group received physiological saline solution (NaCl 0.9%) intraperitoneally (ip) for 7 consecutive days, starting on the third day. The volume of solution injected was equal to the volume of gentamicin delivered to the other groups.

Rats in the other three groups received gentamicin (Gentamicina 80 mg/2 mL, KRKA D.D., Novo mesto, Slovenia), ip, in a single dose of 60 mg/kg/day, daily for 7 successive days, starting with the third day.

The animals in the curcumin group received 230 mg/kg/day of curcumin (Curcumin 95 C3 Complex, Herbagetica, Romania; 1 capsule contains 380 mg of standardized extract of *Curcuma longa* 64–66:1, 20 mg of *Piper nigrum*) dissolved in sunflower seed oil (2 mL).

The animals in the vitamin C group received 200 mg/kg/day of vitamin C (Vitamina C 750 mg, Arena, Romania) diluted in physiological saline (2 mL).

Curcumin and vitamin C were administered orally, using an orogastric tube, daily, starting from the first day, for a total of 9 consecutive days, to assess whether they can prevent the nephrotoxic effects of gentamicin.

The rats were placed in metabolic cages to collect urine for a period of 24 h (day 8/9, day 9/10). Because the number of metabolic cages was insufficient, we collected urine from a limited number of animals: 2 rats from the control group and 4 rats from each of the other groups.

Blood was collected by retroorbital puncture 24 h after the last gentamicin injection (day 10) under general anesthesia. The serum was stored at −20 °C until further analysis. Insufficient blood was obtained from two rats in the vitamin C group; therefore, one sample was used for biochemical analysis and the other sample for ELISA (n = 6). One rat from the curcumin group died on the second day for unknown reasons and was not replaced (n = 6). The remaining rats were sacrificed by cervical dislocation after blood was collected.

### 2.3. Renal Function Evaluation

We determined serum urea and creatinine levels using commercially available kits (Urea-LQ, Creatinine-J; Spinreact, Spain). We followed the kit’s instructions precisely.

### 2.4. Oxidative Stress Analysis

We determined serum levels of malondialdehyde (MDA), nitric oxide (NO), 3-nitrotyrosine (3-NT) and total oxidative stress (TOS).

Serum MDA levels were determined using a technique reported by Mitev et al. [20], with values expressed as MDA nmol/l.

Serum NO concentrations were measured using an assay described by Miranda et al. [21], by standard nitrate reduction and detection by VCl3/Griess assay.

Serum 3-NT levels were determined using a 3-NT (3-Nitrotyrosine) ELISA Kit (E-EL-0040), Elabscience^®^. We respected the instructions from the kit precisely.

Serum concentrations of total oxidative stress were measured using the technique described by Erel [22].

### 2.5. Antioxidant and Anti-Inflammatory Analysis

We determined serum levels of interleukin-10 (IL-10), thiols and total antioxidant capacity (TAC).

Serum IL-10 levels were measured using the Rat IL-10 (Interleukin 10) ELISA Kit (E-EL-R0016), Elabscience^®^. We respected the kit’s directions precisely.

We determined the serum concentrations of thiols using a method designed by Hu [23].

Serum TAC levels were measured using an automatic measurement method developed by Erel [24]. The results were expressed in mmol Trolox/L equivalent.

### 2.6. Histological Analysis

Post-mortem, both kidneys were harvested from each animal and fixed in 10% formalin solution, neutral pH, for 24–48 h. The samples were processed using the standard paraffin inclusion protocol, finally obtaining paraffin blocks. These were sectioned with the manual microtome Thermo Scientific HM325, the resulting sections having a thickness of about 2–3 μm. These sections were displayed on histological slides at 58 °C for 24 h, after which they were stained with hematoxylin-eosin (H&E). An Olympus BX4 microscope was used to examine the obtained preparations, and the Olympus UC30 camera and Stream Basic software were used to take the images.

Samples were evaluated semi-quantitatively using grading scales according to Al-Shabanah et al. [25] and Melnikov et al. [26]. The following parameters were considered for noting the lesions:

- Overall damage to the parenchyma—0: unaffected parenchyma; 1: ≤10% of the affected parenchyma; 2: 11–25% of the affected parenchyma; 3: 26–45% of the affected parenchyma; 4: 46–75% of the affected parenchyma; 5: >75% of the affected parenchyma;
- Glomerular lesions—0: absence of lesions; 1: ≤10% glomeruli with non-specific lesions; 2: 11–25% glomeruli with non-specific lesions; 3: 26–45% glomeruli with non-specific lesions; 4: 46–75% glomeruli with non-specific lesions; 5: >75% glomeruli with non-specific lesions;
- Acute tubular necrosis—0: absent tubular necrosis; 1: ≤10% of the affected tubes; 2: 11–25% of the affected tubes; 3: 26–45% of the affected tubes; 4: 46–75% of the affected tubes; 5: >75% of the affected tubes;
- Tubulo-intestinal inflammatory infiltrate—0: lack of inflammatory infiltrate; 1: discrete inflammatory infiltrate at the interstitial level; 2: severe inflammatory infiltrate both at the interstitium and at the tubular level.

### 2.7. Statistical Analysis

Normality was assessed using the Kolmogorov–Smirnov test. For data with a normal distribution, the results are presented as mean values ± standard error (SE). Weights were compared using the Student’s paired *t*-test. Additionally, for group comparison, one-way ANOVA with post hoc Tukey’s test was performed. For non-parametric data, the results are presented as median values ± interquartile range (IQR). For comparison of these data, Kruskal-Wallis with post hoc Dunn’s test was used.

## 3. Results

The animals had a 100% survival rate in the following groups: control, gentamicin and vitamin C. One rat in the curcumin group died on the third day and was not replaced.

The body weight of all animals who received gentamicin decreased significantly throughout the experiment, while the animals in the control group significantly gained weight, as shown in Table 1.

Diuresis was significantly decreased in all study groups compared to the control (Table 2). The result of the ANOVA test was *p* = 0.0002 and the following group differences were observed: gentamicin vs. control *p* = 0.0002, curcumin vs. control *p* = 0.0036, vitamin C vs. control *p* = 0.0005.

**Figure 1:**
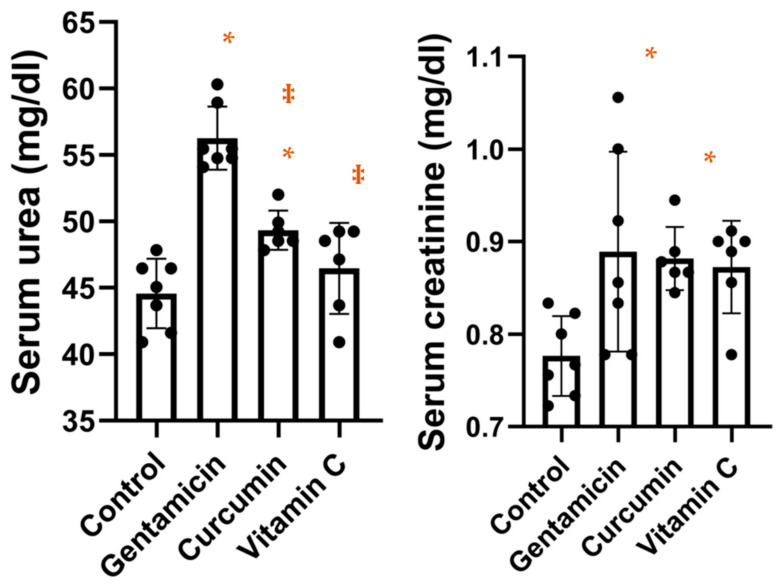
Box-plot showing serum urea and creatinine concentrations in our study groups (* *p* < 0.05 vs. control; ‡ *p* < 0.05 vs. gentamicin).

As shown in Figure 1, serum urea levels were significantly higher in the gentamicin group (*p* < 0.0001) and the curcumin group (*p* = 0.0145) compared to the control group, while no differences were observed between the vitamin C group compared to the control group. Additionally, urea levels were significantly lower in the curcumin group (*p* = 0.0004) and the vitamin C group (*p* < 0.0001) compared to the gentamicin group. The result of the ANOVA test was *p* < 0.0001. For serum creatinine levels, the result of the ANOVA test was *p* = 0.0168. As shown in Figure 1, creatinine was significantly higher in the gentamicin group compared to the control group (*p* = 0.0233) and in the curcumin group compared to the control group (*p* = 0.0465).

**Figure 2:**
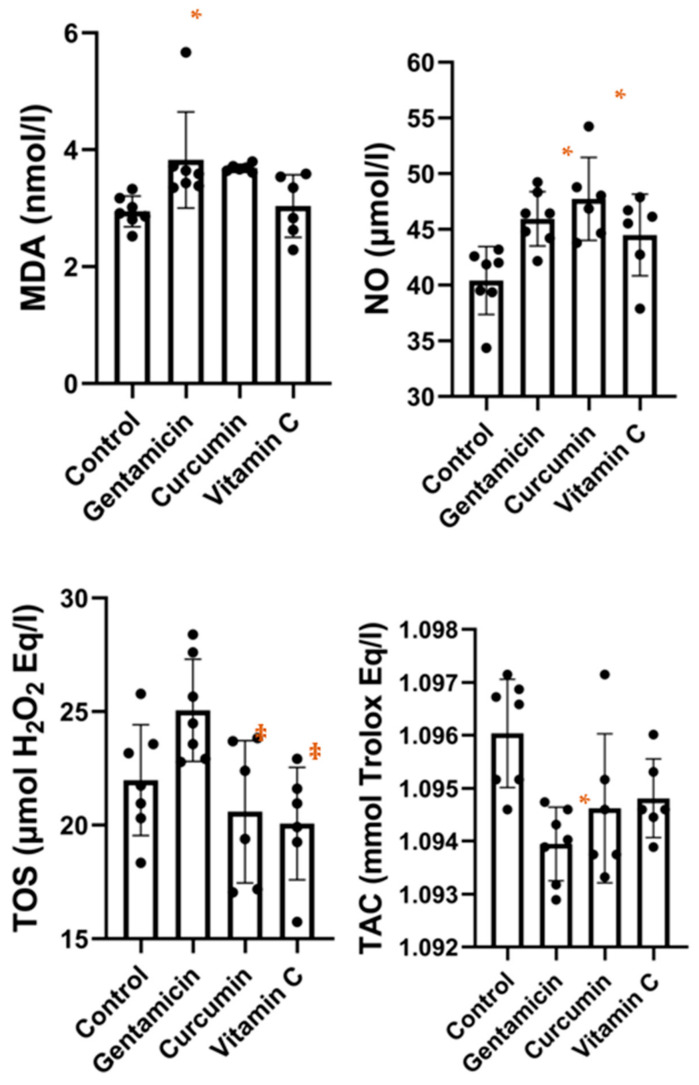
Box-plot displaying serum malondialdehyde, nitric oxide, total oxidative stress, and total antioxidant capacity concentrations in our study groups (* *p* < 0.05 vs. control; ‡ *p* < 0.05 vs. gentamicin).

Figure 2 illustrates serum levels of MDA, NO, TOS and TAC. Serum MDA levels were significantly higher in the gentamicin group compared to the control group (*p* = 0.0212). No other significant differences were found for MDA between the groups. The result of the ANOVA test was *p* = 0.0091. Serum NO concentrations were significantly higher in the gentamicin group (*p* = 0.0192) and the curcumin group (*p* = 0.0025) compared to the control, while no significant differences were found for the vitamin C group compared to any of the other groups. The result of the ANOVA test was *p* = 0.0031. For serum TOS levels, the result of the ANOVA test was *p* = 0.0083. TOS levels were significantly higher in the gentamicin group (*p* = 0.0237) compared to the curcumin group and also compared to the vitamin C group (*p* = 0.01041). The total antioxidant capacity was significantly lower in the gentamicin group (*p* = 0.0038) compared to the control. The result of the ANOVA test was *p* = 0.0066. No other significant differences were found between the groups.

**Figure 3:**
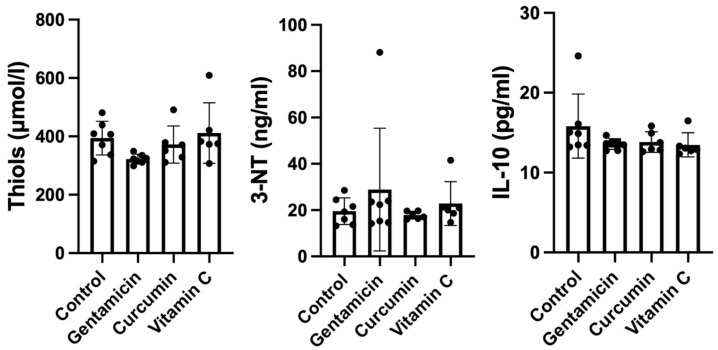
Box-plot showing serum levels of thiols, 3-nitrotyrosine and interleukin-10 in our study groups (*p* > 0.05).

No significant differences were found for thiols (ANOVA *p* = 0.1062), 3-NT (ANOVA *p* = 0.551) and IL-10 (ANOVA *p* = 0.2321) between our study groups (Figure 3).

**Figure 4:**
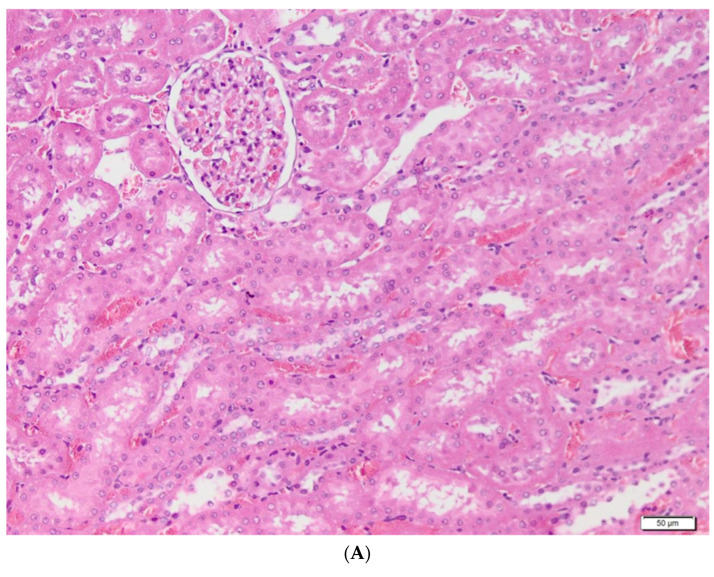

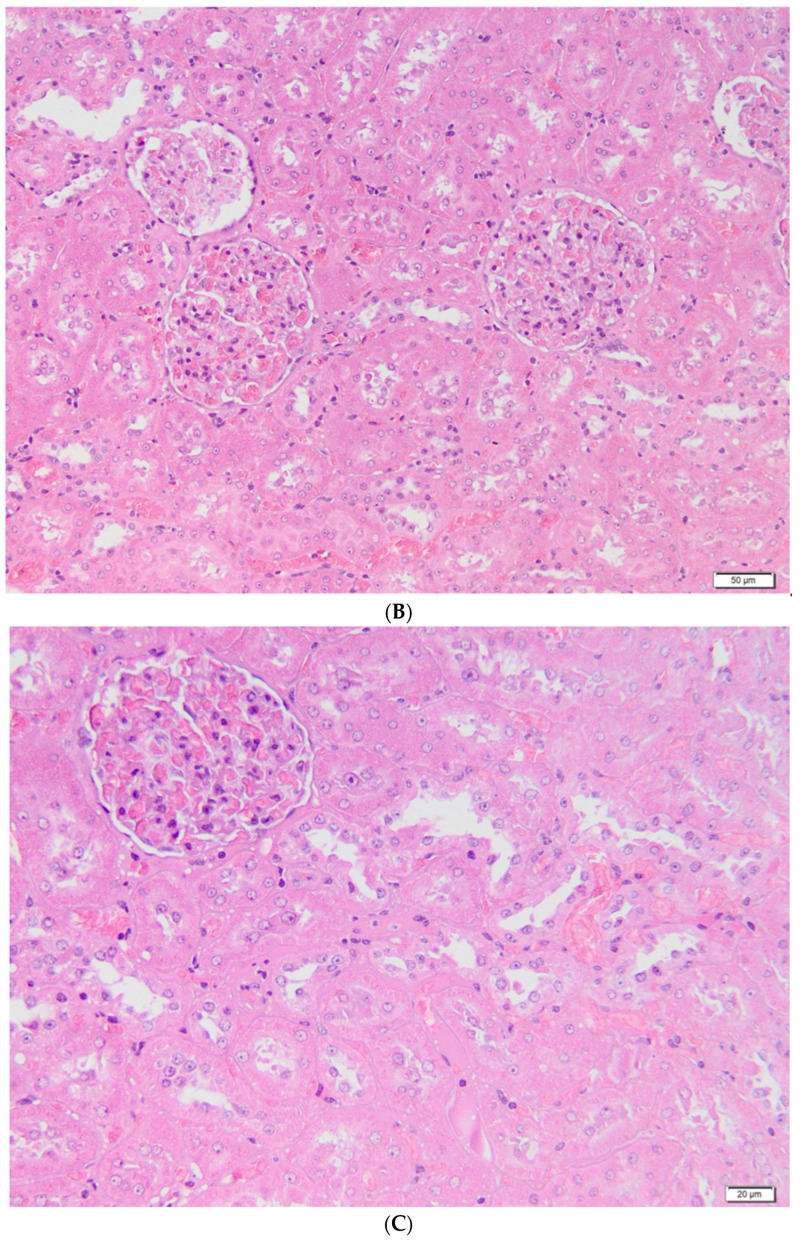

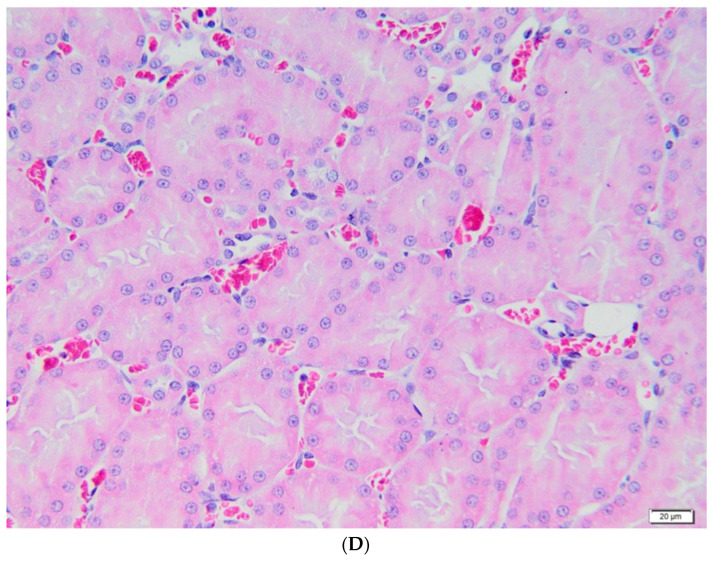
Control group—histological aspects. (**A**–**C**)—Renal cortex, including the unmodified morphological aspects of the tubules, glomeruli and the interstitium; (**D**)—Renal tubules made up of cuboidal cells, with a brush border, showing intracytoplasmic fine granulations, acidophilic, located toward the basal pole of the cells, specific to the rat and without pathological significance.

The control group served as a negative control in the study. All the components of the nephron were evaluated: the renal corpuscle consisting of the glomerulus, with a size of approximately 90 µm and the Bowmann capsule, the renal tubules, consisting of cuboidal epithelial cells with a brush border (microvilli), arranged on a basement membrane, as well as the renal interstitium. From a histological point of view, no changes were observed. The normal structure of the renal parenchyma in our rats is shown in Figure 4.

**Figure 5:**
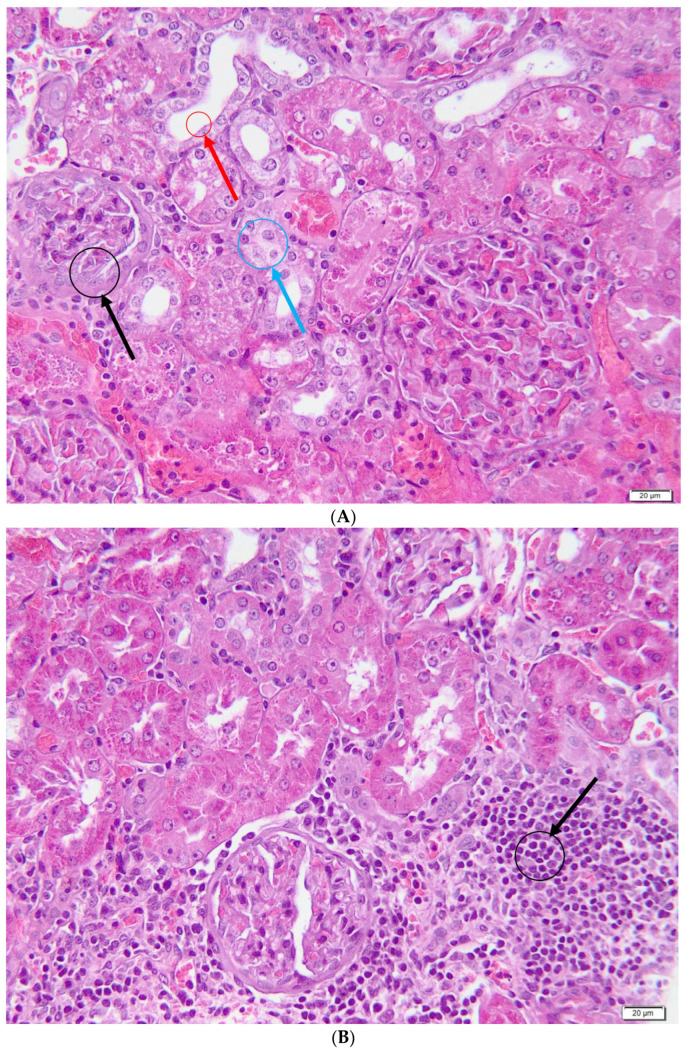

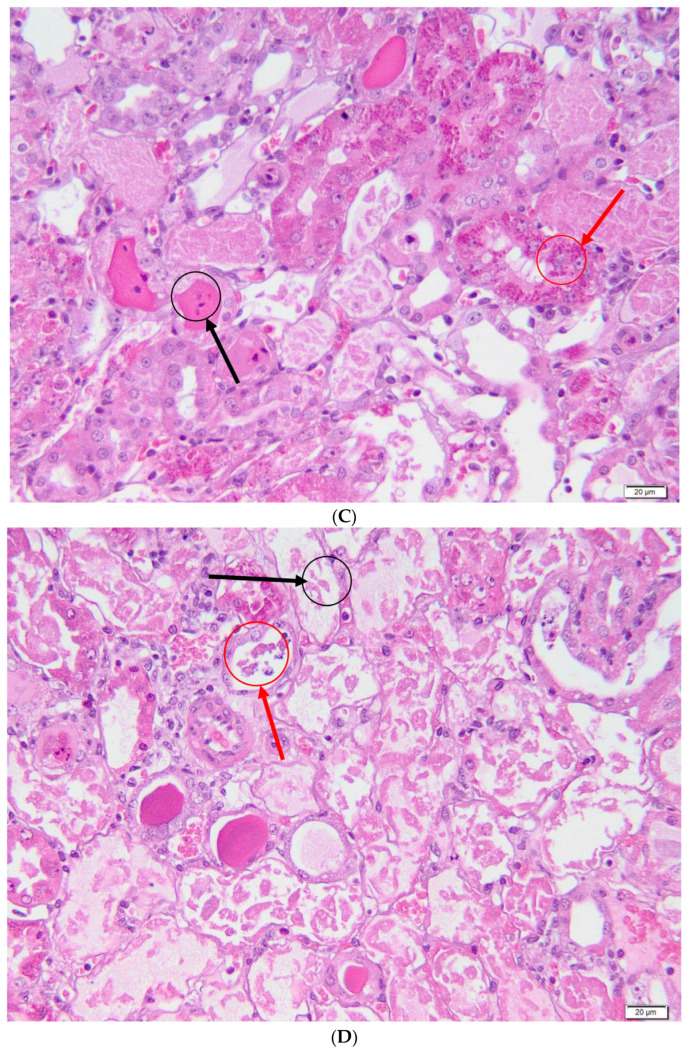
Gentamicin group—histological aspects. (**A**)—Loss of the brush edge of the renal epithelial cells (red arrow), associated with dilation of the lumen of the renal tubules, hydropic degeneration (blue arrow), thickening of the Bowmann capsule and glomerular atrophy (black arrow); (**B**)—Severe multifocal tubulo-interstitial nephritis with extension in the periglomerular area; (**C**)—Extracellular and intracellular hyalinosis (red arrow), with the formation of hyaline cylinders (black arrow); (**D**)—Renal tubular necrosis (black arrow), focal-extensive, acute, severe with the presence of numerous cellular detritus in the renal tubules and thickening of the basement membrane (red arrow).

In the gentamicin group, a series of lesions with pathological significance was highlighted, as shown in Figure 5. Acute tubular necrosis was characterized by aspects of pyknosis, fragmentation of nuclei, detachment of cells from the basement membrane and their individualization, together with cellular detritus in the lumen of the renal tubules. Intracytoplasmically, the presence of a granular, hyperacidophilic material, represented by hyaline bodies (intracellular hyalinosis), was also observed at the level of the renal tubules. Multifocally, the renal tubules were distended due to the presence of a homogeneous/fine granular acidophilic material (extracellular hyalinosis), which determined the compression atrophy of the renal epithelial cells. Diffuse, epithelial cell cytoplasm showed varying degrees of cytoplasmic vacuolization (hydropic degeneration). At the level of the renal corpuscle, multifocal thickening of Bowmann’s capsule and atrophy of the renal glomeruli were observed in all evaluated individuals. Regarding the renal interstitium, a multifocal moderate inflammatory infiltrate composed of neutrophils, lymphocytes and macrophages was observed. All of these changes are part of the picture of acute renal failure.

**Figure 6:**
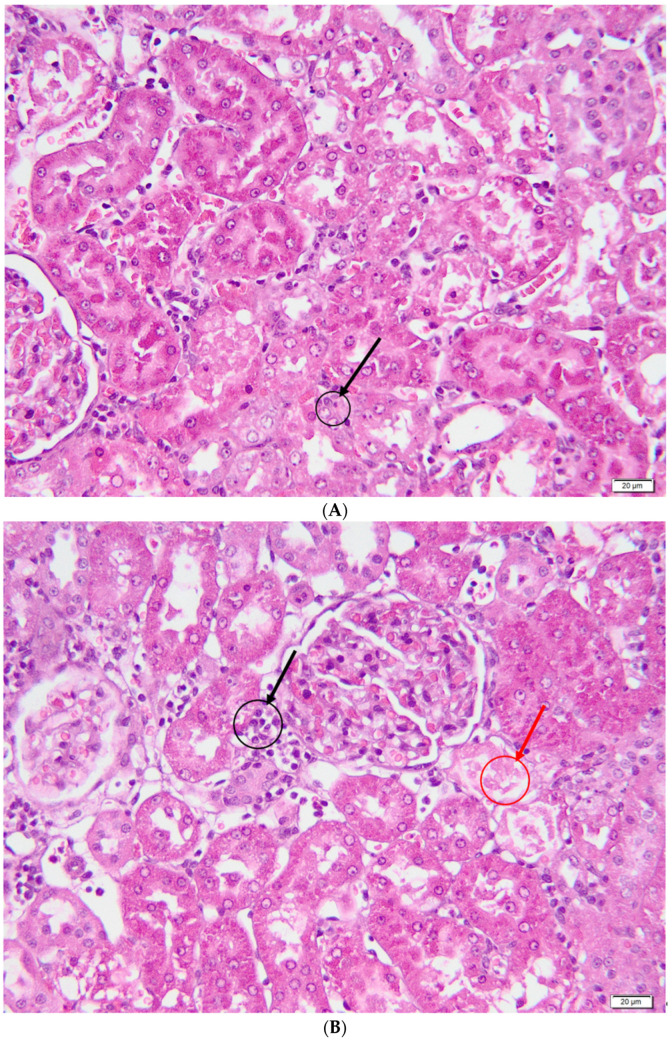

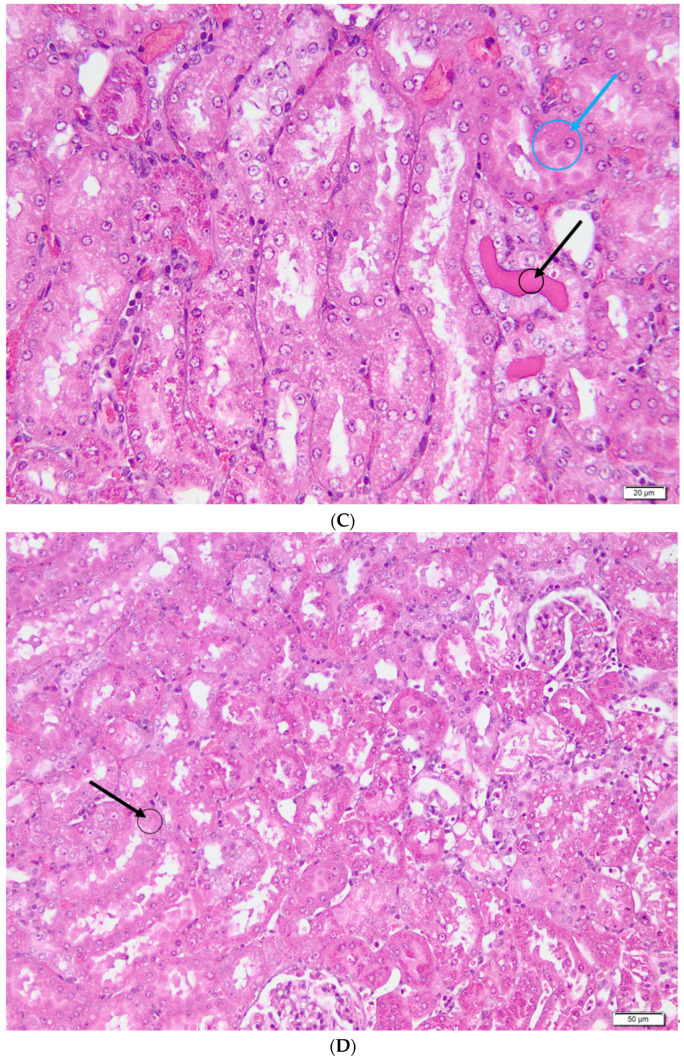
Vitamin C group—histological aspects. (**A**)—Tubular epithelial cells with pycnotic nuclei (caryorexia); (**B**)—Discrete interstitial inflammatory infiltrate (black arrow) and rare aspects of necrosis (red arrow); (**C**)—Intracellular (blue arrow) and extracellular (black arrow) hyalinosis; (**D**)—Discrete hydropic degeneration.

In the vitamin C group (Figure 6), renal tubular necrosis showed a multifocal distribution, and the affected areas were small in size. Hydropic degeneration was observed multifocally in the epithelial cells of the proximal tubules but with a lower degree of severity. Discrete aspects of tubulo-interstitial nephritis, intracellular and extracellular hyalinosis, were also multifocally present.

**Figure 7:**
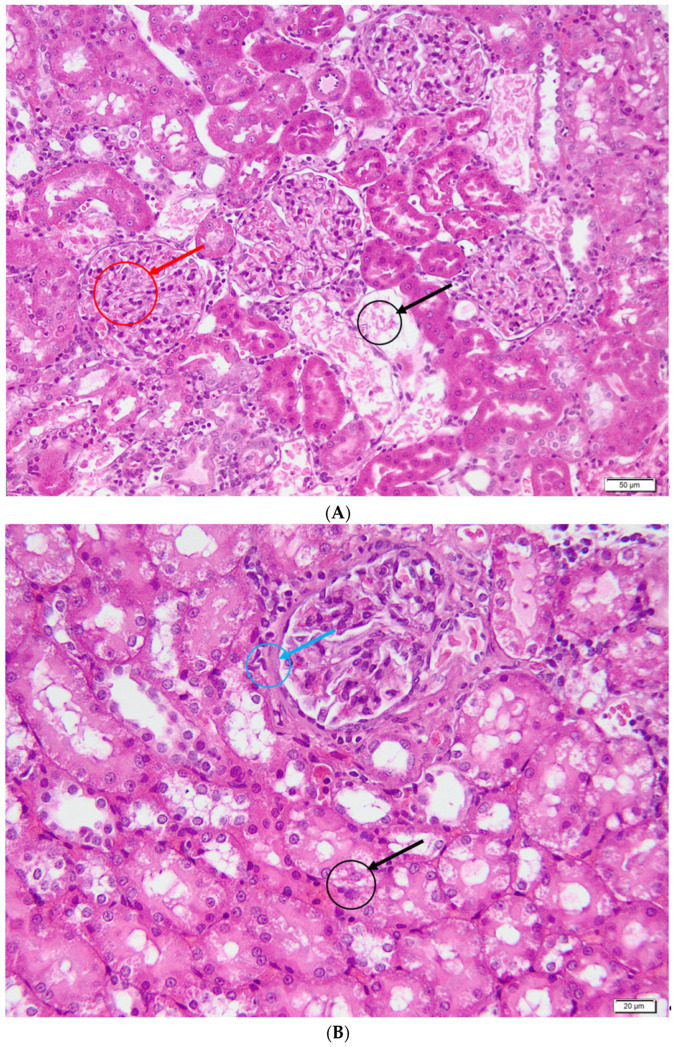

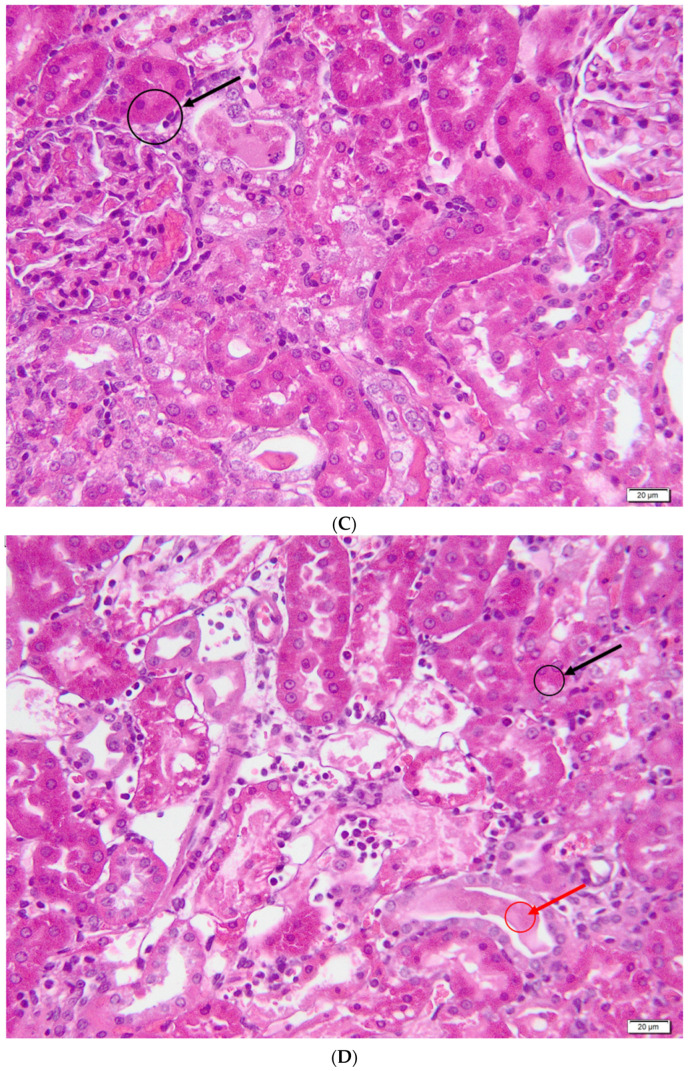
Curcumin group—histological aspects. (**A**)—Acute renal tubular necrosis (black arrow) and hypercellularity at the glomerular level (red arrow); (**B**)—Bowmann capsule thickening (blue arrow) and hydropic, multifocal, moderate degeneration (black arrow); (**C**)—Tubulo-interstitial nephritis, multifocal, discrete (arrow); (**D**)—Intracellular (black arrow) and extracellular (red arrow) hyalinosis associated with an area of renal tubular epithelial necrosis.

In the curcumin group (Figure 7), the areas of tubular necrosis were multifocal, and they represented a smaller percentage of the evaluated renal parenchyma. At the level of renal epithelial cells, degenerative-type lesions were represented by hydropic degeneration and intracellular and extracellular hyalinosis. Bowmann’s capsule thickening and glomerular hypercellularity were observed in the renal corpuscles. Interstitial nephritis was present in all individuals in this group, with low/moderate intensity.

Therefore, all animals treated with gentamicin showed histopathological changes consistent with acute renal injury: acute tubular necrosis, intracellular and extracellular hyaline, hydropic degeneration, thickening of the Bowmann capsule, atrophy of the renal glomeruli, and inflammatory infiltrate. No pathological changes were observed in the control group. The following variables were evaluated for each group: the degree of parenchymal lesion, the severity of glomerular lesions, the degree of acute tubular necrosis and the presence of tubulo-interstitial inflammatory infiltrate.

The statistical results for comparison between the groups are summarized in Table 3. There were significant differences between the control group and the gentamicin group, confirming the successful induction of nephrotoxicity. Compared to the gentamicin group, rats supplemented with vitamin C had significantly lighter lesions alone in all respects considered. In addition, animals supplemented with curcumin had significantly lower scores in terms of glomerular lesions compared to the gentamicin group. Moreover, there were significant differences between the curcumin group and the vitamin C group regarding acute tubular necrosis (*p* = 0.0015) and parenchymal damage (*p* = 0.001).

## 4. Discussion

Approved for medical use more than half a century ago, gentamicin remains a widely used antibiotic for the treatment of severe Gram-negative infections in all age groups, despite its well-known nephrotoxic effects [27,28]. Numerous studies have focused on deciphering the exact mechanisms that lead to this unfavorable outcome, concluding that gentamicin accumulates in proximal tubular cells. From there, gentamicin is internalized by endocytosis and transported to lysosomes, where it binds to their membrane, causing phospholipidosis. Once released into the cytoplasm, gentamicin acts on mitochondria, leading to oxidative stress, activation of the intrinsic apoptosis pathway and cell death, subsequently impairing renal function. The discovery of this gentamicin-induced nephrotoxicity pathway has led to many theories that specific antioxidants can be used to combat it [29,30,31].

Most similar studies focused on the induction of acute kidney injury using gentamicin, disregarding the initial stages in which nephrotoxicity may go undetected, although that is usually the case in clinical practice. Typically, in most of these studies, a high dose of gentamicin (100–150 mg/kg/day) is used, and the typical outcome is a severe form of acute kidney injury (AKI) [32]. The variation in serum creatinine, corroborated by impaired urine output, represents the current criteria for AKI. Nonetheless, it is well known that urea levels vary in a flow-dependent manner; therefore, its serum concentration spikes when diuresis is reduced [33]. In contrast, creatinine levels increase when renal function has been compromised by up to 25–50%, suggesting that the degree of kidney damage may be underestimated when using this marker alone [34]. In addition to these classic biomarkers, the newest ones have shown promising results for the diagnosis of early-stage kidney injury: cystatin C, KIM-1, NAG, NGAL, β2-microglobulin, osteopontin, TIMP-1, GST-α, without being implemented in clinical practice [35]. Despite all the available data, no standardized adjuvant therapies have been established to prevent impaired renal function in patients exposed to gentamicin.

To address these disadvantages, we selected a low dose of gentamicin and successfully induced only a mild form of kidney injury, as previously described [36], thus avoiding the undesired systemic side effects of AKI that could potentially alter the results. Compared to the control group, animals exposed exclusively to gentamicin had significantly higher levels of both creatinine and urea, as well as significantly reduced diuresis, as supported by a study conducted by Al-Kuraishy et al. [37]. However, the fact that creatinine increased less than 1.5 times confirms that our animals did not develop AKI. Rats supplemented with curcumin had significantly higher levels of creatinine compared to the control group, and no differences were observed for the vitamin C group compared to the control group. Curcumin successfully attenuated the impact of gentamicin, as shown by the significantly lower levels of urea compared to the gentamicin group, but these levels were still significantly higher than in the control group. However, vitamin C significantly lowered urea levels compared to the gentamicin group, while no differences were observed compared to the control group. These results indicate that curcumin and vitamin C supplements prevent renal function from declining after exposure to gentamicin, especially in terms of urea clearance. One reason for these discrepancies may reside in the fact that we used lower doses of curcumin and vitamin C compared to other experimental studies in which they received up to 400 mg/kg [32,38], short duration of study or small sample size. In addition, our rats were premedicated only two days before gentamicin administration, which is comparable to what happens in the clinical setting, unlike similar studies where premedication is a minimum of 5 days [32].

A well-documented side effect of gentamicin is weight loss [39]. This side effect was also observed at the end of our study, where significant changes in body weight were detected in all our groups, comparable to Al-Kuraishy et al., who also described important weight loss due to treatment with gentamicin [37]. While the control group gained weight significantly over the course of the experiment, all animals in the other groups that were exposed to gentamicin lost weight significantly. Interestingly, supplimenting with curcumin and vitamin C did not prevent their weight loss, despite preserving renal function.

Even though we used low doses of gentamicin, our research still validates the oxidative stress-related mechanism of its nephrotoxic effects, as described in previous research [3,6,7]. Furthermore, the antioxidant effects of curcumin and vitamin C were also validated in our study, as the supplemented rats had significantly lower levels of total oxidative stress compared to the gentamicin group. Our results are in line with previous studies proving their antioxidant capacity [11,14,17].

In this context, we identified two distinct markers of oxidative stress that increased significantly after gentamicin administration, which further validate the oxidative stress-related mechanism: malondialdehyde and nitric oxide. MDA is a reliable marker of oxidative stress and represents a product of lipid peroxidation that indicates an augmented generation of free radicals [38,40]. Similar to what Al-Kuraishy reported [37], in the present study, serum MDA levels were significantly higher in the gentamicin group compared to those of the control group, confirming the involvement of oxidative stress. Supplementation with vitamin C and curcumin maintained MDA levels similar to those in the control group, proving that both substances were able to modulate the serum levels of this marker. Similar results were reported by Laorodphun et al., who showed that curcumin is able to prevent gentamicin-induced nephrotoxicity by preserving normal MDA levels in the renal cortex [41]. Yarijani et al. obtained similar results in decreasing oxidative stress (by reducing MDA levels) by administering *Malva sylvestris* extract in a similar context to gentamicin renal toxicity [42]. Another study showed that curcumin was able to decrease inducible NO synthase, along with the production of reactive oxygen species by mitochondria. Because oxidative stress and mitochondria are strongly interconnected, intracellular signaling to trigger apoptosis can be reduced by modulating oxidative stress and improving mitochondrial function [43].

Additionally, gentamicin induced higher levels of nitric oxide compared to the control group. Serum NO levels increase after exposure to increased concentrations of other reactive species or after direct exposure to an inflammatory stimulus [40]. Curcumin had no effect on preventing an increase in NO levels, while vitamin C seemed to have a slightly positive effect. Additionally, no changes were observed for 3-NT levels, which is in contrast to other reported studies [44], probably due to our reduced doses of gentamicin. Nevertheless, the fact that NO and MDA were detectable in the serum in the early stages of kidney injury suggests that they could be used as markers of gentamicin nephrotoxicity.

Our research highlighted the presence of two main differences between the mechanisms of action of curcumin and vitamin C as antioxidants. As described above, both curcumin and vitamin C successfully reduced TOS levels below baseline but did so by targeting different reactive species. Vitamin C maintained both MDA and NO at levels similar to the control group, while curcumin could not maintain NO at low levels. The present results are consistent with those of Gheith et al. [45], who obtained decreased levels of MDA in animals treated with gentamicin and vitamin C compared to gentamicin alone. Encouragingly, MDA has been effectively counteracted by other natural substances, such as *Solanum xanthocarpum* fruit extract [46]. However, neither curcumin nor vitamin C had any significant effect on serum IL-10, thiols and TAC levels, which could be explained by the small doses administered. The dose-related effects need further investigation. Nevertheless, other studies have reported that pre-treatment with other substances, such as dipyridamole, has successfully prevented the decrease in IL-10 levels [47]. However, the importance of reducing oxidative stress and inflammation is crucial because it translates into a decreased apoptotic and autophagic process, leading to an amelioration in kidney cell survival to toxicant-induced injury [48].

Corroboration reduced TOS, NO and MDA levels with improved serum urea and creatinine levels suggests that both curcumin and vitamin C have nephroprotective effects based on their antioxidant properties.

From a histopathological point of view, there were significant differences between the control group and the gentamicin group, suggesting the successful induction of acute nephrotoxicity. Histological evaluation of the kidneys in animals supplemented with curcumin and vitamin C suggests a reduction in the nephrotoxic effect of gentamicin on renal tubular epithelial cells. Both the curcumin group and the vitamin C group showed more discrete lesions compared to the gentamicin group. These were represented by degenerative lesions and tubular necrosis, with a smaller proportion of cells observed in different stages of necrosis (pycnotic nuclei, karyorexis, karyolysis, cell swelling, and destruction of the cytoplasmic membrane). However, all histological scores were significantly lower in the vitamin C group compared to the gentamicin group, demonstrating its nephroprotective effect. Additionally, the scores obtained in the curcumin group were lower than those in the gentamicin group but only for glomerular lesions. Vitamin C had better results in all aspects considered compared to the gentamicin group. Moreover, it was more beneficial than curcumin, as shown by the significantly lower scores for acute tubular necrosis and parenchymal damage. Similar results were obtained by Stojiljkovic et al., who showed that rats treated with gentamicin and vitamin C underwent degenerative changes in the proximal tubules but without aspects of necrosis, in contrast to the gentamicin group, which underwent segmental necrosis and detachment of epithelial cells [49]. Mahmoud et al. obtained comparable results, as their curcumin-supplemented rats experienced less severe changes in kidney tissue compared to the gentamicin group [50].

One fragile point of our study is that we were unable to determine the glomerular filtration rate due to the limited number of metabolic cages for urine collection. We also missed the curcumin-only group and the vitamin C-only group, a decision based on the need to reduce the number of animals sacrificed. Another weakness of our study is that one rat from the curcumin died unexpectedly, along with insufficient blood collection from two rats in the vitamin C group.

However, unlike most previously published studies, we have focused on the variation of oxidative stress markers in a pre-AKI stage, which is more relevant to what usually happens in clinical practice when mild nephrotoxicity goes unnoticed. We also detected these changes in serum, as we would do in a patient, and not in the kidney tissue homogenate, as is usually done in experimental studies. Furthermore, both curcumin and vitamin C are rather safe, inexpensive and very accessible worldwide. To the best of our knowledge, limited research has been conducted to evaluate their nephroprotective effects. In the future, we will consider conducting further investigations to determine their concurrent effects in vivo. Furthermore, we aim to validate MDA and NO as markers of gentamicin-induced nephrotoxicity in human subjects.

## 5. Conclusions

In the present study, we aimed to determine whether curcumin and vitamin C have nephroprotective effects in a mild form of renal injury induced by gentamicin.

Our results show that curcumin and vitamin C effectively reduced total oxidative stress compared to the gentamicin group by modulating different reactive species. Moreover, we identified two reactive species (malondialdehyde and nitric oxide) that were successfully prevented from rising with vitamin C, suggesting that they could be used as serum markers of the early nephrotoxicity of gentamicin in vivo.

From a histological point of view, vitamin C proved to be more beneficial compared to curcumin, as it successfully limited all unfavorable effects of gentamicin on renal tissue.

Corroborating these data with their effect on reducing urea levels, we concluded that both curcumin and vitamin C have nephroprotective effects due to their antioxidant capacity.

Our results encourage further research to identify the minimum doses required to achieve these favorable effects in humans and to evaluate the effects of their simultaneous administration.

## Figures and Tables

**Table 1:** Weight of the animals included in the study.

		Mean Values ± SD	Student’s *t*-Test
Control group	Initial body weight (g)	383.57 ± 44.87	*p* = 0.0347
	Final body weight (g)	388.57 ± 47.60	
Gentamicin group	Initial body weight (g)	416.57 ± 64.47	*p* = 0.0011
	Final body weight (g)	392.43 ± 66.76	
Curcumin group	Initial body weight (g)	402.17 ± 23.25	*p* = 0.0102
	Final body weight (g)	374.33 ± 39.55	
Vitamin C group	Initial body weight (g)	382.71 ± 16.71	*p* < 0.0001
	Final body weight (g)	340.57 ± 13.53	

SD—standard deviation

**Table 2:** Diuresis in our study groups.

Diuresis (mL/24 h)	Mean Values ± SD	ANOVA	*p* Value, Compared to Control
Control group	21.500 ± 0.707	0.0002	-
Gentamicin group	11.000 ± 1.581		*p* = 0.0002
Curcumin group	14.250 ± 2.217		*p* = 0.0036
Vitamin C group	12.125 ± 1.652		*p* = 0.0005

**Table 3:** Histological scores of renal lesions.

		Control Group	Gentamicin Group	Curcumin Group	Vitamin C Group
Total score	Median	0	7	5.5	3 (* *p* < 0.0001) (^ *p* = 0.0131)
	IQR	0	1	1.75	1.5
Parenchymal damage score	Median	0	4	3	1
	IQR	0	0	0.75	1 (* *p* < 0.0001) (^ *p* = 0.0077)
Glomeruli injury score	Median	0	2	1.5 (* *p* = 0.0305)	1 (* *p* = 0.0001)
	IQR	0	0	1	0
Acute tubular necrosis score	Median	0	3	3	1 (* *p* = 0.0003) (^ *p* = 0.0055)
	IQR	0	1	0	1
Tubulo-interstitial infiltrate score	Median	0	2	1	1 (* *p* = 0.0016)
	IQR	0	0.5	0.75	0.5

Results are presented as median values ± interquartile range (IQR). * *p* compared to the gentamicin group. ^ *p* compared to the curcumin group

## Data Availability

Study data available from the corresponding author. The data is not publicly available because it is part of a doctoral thesis and can be made public only after the publication of the associated thesis.
